# Tricyclo­hexyl[2-(2,3-dimethyl­anilino)benzoato-κ*O*]tin(IV)

**DOI:** 10.1107/S1600536809048314

**Published:** 2009-11-21

**Authors:** Muhammad Danish, M. Nawaz Tahir, Nazir Ahmad, Saqib Ali, Amin Badshah

**Affiliations:** a Department of Chemistry, University of Sargodha, Sargodha, Pakistan; b Department of Physics, University of Sargodha, Sargodha, Pakistan; c Department of Chemistry, Quaid-i-Azam University, Islamabad 45320, Pakistan

## Abstract

In the title compound, [Sn(C_6_H_11_)_3_(C_15_H_14_NO_2_)], the Sn^IV^ atom adopts a distorted tetra­hedral SnOC_3_ arrangement. The dihedral angle between the benzene rings in the mefanamic acid mol­ecule is 82.16 (17)° and intra­molecular N—H⋯O and C—H⋯O hydrogen bonds help to establish the conformation. Two of the cyclo­hexyl rings are disordered over two sets of sites with equal occupancies.

## Related literature

For the synthesis, see: Danish *et al.* (1997 ▶). For related structures, see: Danish *et al.* (1997 ▶, 2009 ▶); Tahir *et al.* (1997*a* ▶,*b* ▶); Willem *et al.* (1998 ▶).
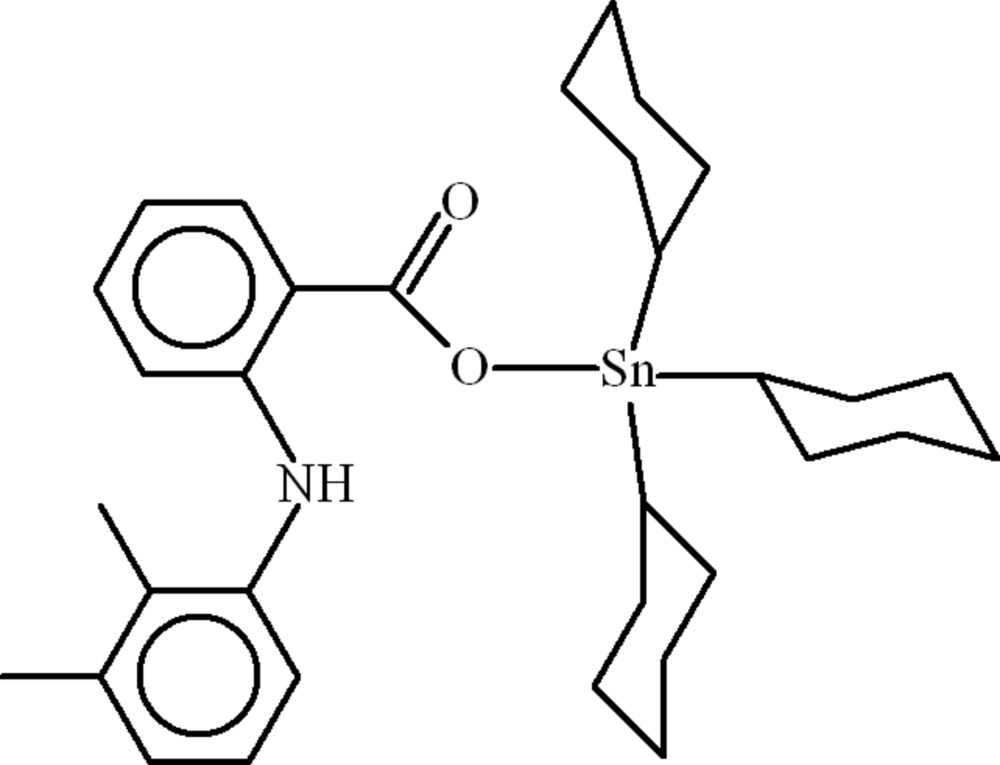


## Experimental

### 

#### Crystal data

[Sn(C_6_H_11_)_3_(C_15_H_14_NO_2_)]
*M* *_r_* = 608.41
Triclinic, 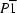

*a* = 9.6093 (2) Å
*b* = 12.0104 (3) Å
*c* = 15.5241 (4) Å
α = 109.872 (1)°
β = 90.616 (2)°
γ = 110.548 (1)°
*V* = 1560.47 (6) Å^3^
*Z* = 2
Mo *K*α radiation
μ = 0.85 mm^−1^
*T* = 296 K
0.28 × 0.22 × 0.20 mm

#### Data collection

Bruker Kappa APEXII CCD diffractometer
Absorption correction: multi-scan (*SADABS*; Bruker, 2005 ▶) *T*_min_ = 0.797, *T*_max_ = 0.841
31924 measured reflections
7684 independent reflections
5308 reflections with *I* > 2σ(*I*)
*R*_int_ = 0.030

#### Refinement

*R*[*F* ^2^ > 2σ(*F* ^2^)] = 0.056
*wR*(*F* ^2^) = 0.166
*S* = 1.02
7684 reflections
307 parameters
33 restraints
H atoms treated by a mixture of independent and constrained refinement
Δρ_max_ = 1.37 e Å^−3^
Δρ_min_ = −0.49 e Å^−3^

### 

Data collection: *APEX2* (Bruker, 2007 ▶); cell refinement: *SAINT* (Bruker, 2007 ▶); data reduction: *SAINT*; program(s) used to solve structure: *SHELXS97* (Sheldrick, 2008 ▶); program(s) used to refine structure: *SHELXL97* (Sheldrick, 2008 ▶); molecular graphics: *ORTEP-3* (Farrugia, 1997 ▶) and *PLATON* (Spek, 2009 ▶); software used to prepare material for publication: *WinGX* (Farrugia, 1999 ▶) and *PLATON*.

## Supplementary Material

Crystal structure: contains datablocks global, I. DOI: 10.1107/S1600536809048314/hb5223sup1.cif

Structure factors: contains datablocks I. DOI: 10.1107/S1600536809048314/hb5223Isup2.hkl

Additional supplementary materials:  crystallographic information; 3D view; checkCIF report

## Figures and Tables

**Table 1:** Selected bond lengths (Å)

Sn—O1	2.073 (3)
Sn—C16	2.130 (6)
Sn—C28	2.147 (5)
Sn—C22	2.153 (6)

**Table 2:** Hydrogen-bond geometry (Å, °)

*D*—H⋯*A*	*D*—H	H⋯*A*	*D*⋯*A*	*D*—H⋯*A*
N1—H1⋯O2	0.90 (7)	1.93 (7)	2.656 (6)	137 (6)
C23*A*—H23*A*⋯O2	0.97	2.45	3.17 (3)	132

**Fig. 1.**
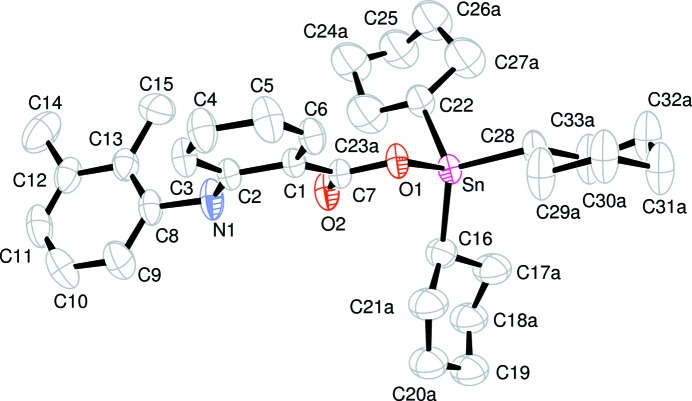
View of (I) with the atom numbering scheme having one part of disordered cyclohexyl rings. The displacement ellipsoids are drawn at the 30% probability level.

**Fig. 2.**
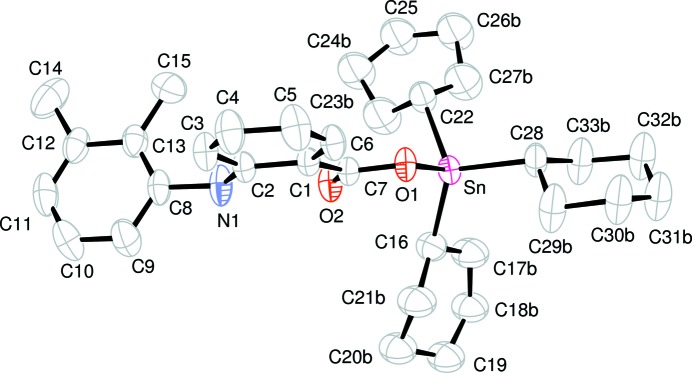
View of (I) with the atom numbering scheme having other part of disordered cyclohexyl rings. The displacement ellipsoids are drawn at the 30% probability level.

## supplementary crystallographic information

### Comment

We reported the crystal structures of (II) {2-[(2,3-Dimethylphenyl)amino]benzoato-*O*:*O*^'^}-trimethyltin(IV) (Tahir *et al.*, 1997*a*), (III) (Ketoprofenato)trimethyltin(IV) (Tahir *et al.*, 1997*b*) and recently (IV) Bis(µ_3_-Oxo)-bis(µ_2_-2- ((3-thiophene)acetato-*O*,*O*^'^)-octa-methyl(2-((3-thiophene)acetato-O)- tetra-tin(IV) (Danish *et al.*, 2009). In continuation of interest with tin chemistry, the title compound (I, Fig. 1) is being reported.

The tricyclohexyltin complexes of carboxylates have tetrahedral coordination such as (2-((*E*)-2-(2-Hydroxy-5-methylphenyl)-1-diazenyl)benzoato)-tricyclohexyl -tin (Willem *et al.*, 1998). The title compound (I) also have distorted tetrahedral coordination. Due to the bulky and twisted ligand of mefenamic acid, the cyclohexyl moieties are disordered. The dihedral angle between the benzene rings A (C1–C6) and B (C8–C13) is 82.16 (17)°. The molecules are stabilized in the form of monomers with two intramolecular H-bondings (Table 1).

### Experimental

The title compound was prepared according to the method described already (Danish *et al.*, 1997) to yield colourless prisms of (I).

### Refinement

The disorder in the cyclohexyl rings occurs as the C–C bond distances become shorter when refined isotropically or anisotropically. The higher peak remains present to the Sn-atom if refined without consideration of disorder also. However, lower *R*-values convinced to submit the crystal structure in the present form.

All the cyclohexyl rings were refined with equal occupancy ratio and using EADP for the individual rings.

The coordinates of H1 attached to N1 were refined. The other H-atoms were positioned geometrically (C–H = 0.93–0.97 Å) and refined as riding with *U*_iso_(H) = 1.2*U*_eq_(carrier) or 1.5*U*_eq_(methyl C).

### Crystal data

**Table:**

[Sn(C_6_H_11_)_3_(C_15_H_14_NO_2_)]	*Z* = 2
*M**_r_* = 608.41	*F*(000) = 636
Triclinic, *P*1	*D*_x_ = 1.298 Mg m^−^^3^
Hall symbol: -P 1	Mo *K*α radiation, λ = 0.71073 Å
*a* = 9.6093 (2) Å	Cell parameters from 7684 reflections
*b* = 12.0104 (3) Å	θ = 2.3–28.3°
*c* = 15.5241 (4) Å	µ = 0.85 mm^−^^1^
α = 109.872 (1)°	*T* = 296 K
β = 90.616 (2)°	Prism, colourless
γ = 110.548 (1)°	0.28 × 0.22 × 0.20 mm
*V* = 1560.47 (6) Å^3^	

### Data collection

**Table:**

Bruker Kappa APEXII CCD diffractometer	7684 independent reflections
Radiation source: fine-focus sealed tube	5308 reflections with *I* > 2σ(*I*)
graphite	*R*_int_ = 0.030
Detector resolution: 7.40 pixels mm^-1^	θ_max_ = 28.3°, θ_min_ = 2.3°
ω scans	*h* = −12→8
Absorption correction: multi-scan (*SADABS*; Bruker, 2005)	*k* = −15→15
*T*_min_ = 0.797, *T*_max_ = 0.841	*l* = −20→20
31924 measured reflections	

### Refinement

**Table:**

Refinement on *F*^2^	Primary atom site location: structure-invariant direct methods
Least-squares matrix: full	Secondary atom site location: difference Fourier map
*R*[*F*^2^ > 2σ(*F*^2^)] = 0.056	Hydrogen site location: inferred from neighbouring sites
*wR*(*F*^2^) = 0.166	H atoms treated by a mixture of independent and constrained refinement
*S* = 1.02	*w* = 1/[σ^2^(*F*_o_^2^) + (0.0862*P*)^2^ + 1.3991*P*] where *P* = (*F*_o_^2^ + 2*F*_c_^2^)/3
7684 reflections	(Δ/σ)_max_ < 0.001
307 parameters	Δρ_max_ = 1.37 e Å^−^^3^
33 restraints	Δρ_min_ = −0.49 e Å^−^^3^

### Special details

**Table:**

Geometry. All e.s.d.'s (except the e.s.d. in the dihedral angle between two l.s. planes) are estimated using the full covariance matrix. The cell e.s.d.'s are taken into account individually in the estimation of e.s.d.'s in distances, angles and torsion angles; correlations between e.s.d.'s in cell parameters are only used when they are defined by crystal symmetry. An approximate (isotropic) treatment of cell e.s.d.'s is used for estimating e.s.d.'s involving l.s. planes.
Refinement. Refinement of *F*^2^ against ALL reflections. The weighted *R*-factor *wR* and goodness of fit *S* are based on *F*^2^, conventional *R*-factors *R* are based on *F*, with *F* set to zero for negative *F*^2^. The threshold expression of *F*^2^ > σ(*F*^2^) is used only for calculating *R*-factors(gt) *etc*. and is not relevant to the choice of reflections for refinement. *R*-factors based on *F*^2^ are statistically about twice as large as those based on *F*, and *R*- factors based on ALL data will be even larger.

### Fractional atomic coordinates and isotropic or equivalent isotropic displacement parameters (Å^2^)

**Table:**

	*x*	*y*	*z*	*U*_iso_*/*U*_eq_	Occ. (<1)
Sn	0.36022 (4)	0.37096 (3)	0.16644 (2)	0.06337 (15)	
O1	0.2926 (4)	0.4860 (3)	0.1161 (2)	0.0701 (8)	
O2	0.3934 (5)	0.6278 (3)	0.2549 (2)	0.0815 (10)	
N1	0.3785 (6)	0.8571 (4)	0.3012 (3)	0.0864 (14)	
H1	0.413 (7)	0.803 (6)	0.314 (5)	0.104*	
C1	0.2665 (5)	0.6839 (4)	0.1546 (3)	0.0565 (10)	
C2	0.2909 (6)	0.8087 (4)	0.2175 (3)	0.0622 (11)	
C3	0.2220 (7)	0.8792 (5)	0.1918 (4)	0.0785 (14)	
H3	0.2350	0.9605	0.2328	0.094*	
C4	0.1360 (8)	0.8315 (6)	0.1077 (4)	0.0930 (18)	
H4	0.0912	0.8809	0.0926	0.112*	
C5	0.1136 (8)	0.7119 (6)	0.0446 (4)	0.097 (2)	
H5	0.0558	0.6808	−0.0131	0.116*	
C6	0.1779 (6)	0.6405 (5)	0.0685 (3)	0.0740 (13)	
H6	0.1627	0.5594	0.0261	0.089*	
C7	0.3235 (5)	0.5990 (4)	0.1789 (3)	0.0604 (10)	
C8	0.3998 (7)	0.9770 (5)	0.3718 (3)	0.0735 (13)	
C9	0.5116 (9)	1.0862 (6)	0.3712 (5)	0.103 (2)	
H9	0.5696	1.0827	0.3235	0.124*	
C10	0.5363 (10)	1.1996 (6)	0.4415 (6)	0.118 (2)	
H10	0.6094	1.2745	0.4409	0.141*	
C11	0.4540 (10)	1.2034 (6)	0.5126 (5)	0.106 (2)	
H11	0.4765	1.2806	0.5619	0.127*	
C12	0.3412 (7)	1.0990 (6)	0.5138 (4)	0.0885 (17)	
C13	0.3121 (6)	0.9806 (5)	0.4406 (4)	0.0790 (14)	
C14	0.2511 (10)	1.1074 (10)	0.5923 (6)	0.147 (4)	
H14A	0.2621	1.1946	0.6240	0.220*	
H14B	0.2864	1.0773	0.6347	0.220*	
H14C	0.1471	1.0556	0.5686	0.220*	
C15	0.1874 (8)	0.8636 (7)	0.4391 (6)	0.116 (2)	
H15A	0.0980	0.8815	0.4500	0.174*	
H15B	0.2139	0.8371	0.4866	0.174*	
H15C	0.1697	0.7966	0.3798	0.174*	
C16	0.5928 (6)	0.4556 (6)	0.2219 (5)	0.0889 (16)	
H16	0.5985	0.5098	0.2864	0.107*	
C19	0.9166 (8)	0.5092 (8)	0.2374 (7)	0.1222 (16)	
H19A	1.0163	0.5592	0.2728	0.147*	
H19B	0.9249	0.4457	0.1812	0.147*	
C17A	0.666 (2)	0.360 (2)	0.227 (2)	0.1222 (16)	0.50
H17A	0.6016	0.2988	0.2509	0.147*	0.50
H17B	0.6830	0.3142	0.1656	0.147*	0.50
C18A	0.8174 (18)	0.4443 (17)	0.2926 (13)	0.1222 (16)	0.50
H18A	0.8035	0.5062	0.3476	0.147*	0.50
H18B	0.8593	0.3918	0.3111	0.147*	0.50
C20A	0.856 (3)	0.594 (2)	0.2134 (18)	0.1222 (16)	0.50
H20A	0.9105	0.6189	0.1666	0.147*	0.50
H20B	0.8791	0.6707	0.2680	0.147*	0.50
C21A	0.687 (2)	0.544 (5)	0.178 (4)	0.1222 (16)	0.50
H21A	0.6568	0.6164	0.1906	0.147*	0.50
H21B	0.6704	0.4997	0.1112	0.147*	0.50
C17B	0.6452 (19)	0.363 (2)	0.237 (2)	0.1222 (16)	0.50
H17C	0.6101	0.2864	0.1815	0.147*	0.50
H17D	0.5954	0.3392	0.2862	0.147*	0.50
C18B	0.8160 (16)	0.3987 (15)	0.2633 (14)	0.1222 (16)	0.50
H18C	0.8381	0.4214	0.3296	0.147*	0.50
H18D	0.8404	0.3243	0.2326	0.147*	0.50
C20B	0.860 (3)	0.615 (2)	0.2389 (18)	0.1222 (16)	0.50
H20C	0.9265	0.6702	0.2111	0.147*	0.50
H20D	0.8580	0.6656	0.3021	0.147*	0.50
C21B	0.701 (2)	0.548 (5)	0.183 (4)	0.1222 (16)	0.50
H21C	0.6621	0.6125	0.1834	0.147*	0.50
H21D	0.7066	0.5014	0.1194	0.147*	0.50
C22	0.2180 (7)	0.3375 (5)	0.2686 (4)	0.0855 (15)	
H22	0.1377	0.3585	0.2470	0.103*	
C25	0.1326 (12)	0.2839 (8)	0.4341 (6)	0.152 (3)	
H25A	0.0785	0.2655	0.4832	0.182*	
H25B	0.2288	0.2752	0.4385	0.182*	
C23A	0.263 (3)	0.441 (3)	0.3636 (15)	0.152 (3)	0.50
H23A	0.2702	0.5215	0.3583	0.182*	0.50
H23B	0.3610	0.4525	0.3900	0.182*	0.50
C24A	0.146 (3)	0.4080 (17)	0.4296 (17)	0.152 (3)	0.50
H24A	0.1786	0.4741	0.4910	0.182*	0.50
H24B	0.0488	0.4029	0.4060	0.182*	0.50
C26A	0.040 (2)	0.207 (2)	0.3386 (10)	0.152 (3)	0.50
H26A	−0.0338	0.2430	0.3327	0.182*	0.50
H26B	−0.0142	0.1207	0.3356	0.182*	0.50
C27A	0.123 (2)	0.1990 (16)	0.2542 (13)	0.152 (3)	0.50
H27A	0.1858	0.1504	0.2513	0.182*	0.50
H27B	0.0520	0.1588	0.1974	0.182*	0.50
C23B	0.307 (2)	0.418 (2)	0.3655 (12)	0.152 (3)	0.50
H23C	0.3851	0.3867	0.3737	0.182*	0.50
H23D	0.3563	0.5053	0.3696	0.182*	0.50
C24B	0.216 (2)	0.4165 (16)	0.4436 (16)	0.152 (3)	0.50
H24C	0.2819	0.4627	0.5025	0.182*	0.50
H24D	0.1465	0.4582	0.4418	0.182*	0.50
C26B	0.094 (3)	0.1719 (17)	0.3434 (11)	0.152 (3)	0.50
H26C	−0.0102	0.1438	0.3175	0.182*	0.50
H26D	0.1099	0.1009	0.3524	0.182*	0.50
C27B	0.199 (3)	0.220 (2)	0.2790 (14)	0.152 (3)	0.50
H27C	0.2975	0.2250	0.2992	0.182*	0.50
H27D	0.1646	0.1533	0.2178	0.182*	0.50
C28	0.2913 (7)	0.2114 (5)	0.0371 (4)	0.0813 (15)	
H28	0.1818	0.1731	0.0324	0.098*	
C29A	0.317 (3)	0.2527 (16)	−0.0469 (10)	0.122 (2)	0.50
H29A	0.2636	0.3084	−0.0453	0.146*	0.50
H29B	0.4233	0.3010	−0.0428	0.146*	0.50
C30A	0.265 (3)	0.1407 (16)	−0.1369 (12)	0.122 (2)	0.50
H30A	0.2995	0.1704	−0.1865	0.146*	0.50
H30B	0.1557	0.1059	−0.1479	0.146*	0.50
C31A	0.318 (3)	0.0348 (17)	−0.1409 (10)	0.122 (2)	0.50
H31A	0.2743	−0.0370	−0.1989	0.146*	0.50
H31B	0.4265	0.0650	−0.1371	0.146*	0.50
C32A	0.273 (3)	−0.0041 (18)	−0.0648 (10)	0.122 (2)	0.50
H32A	0.3045	−0.0733	−0.0672	0.146*	0.50
H32B	0.1643	−0.0355	−0.0699	0.146*	0.50
C33A	0.341 (3)	0.1060 (18)	0.0271 (11)	0.122 (2)	0.50
H33A	0.3130	0.0755	0.0772	0.146*	0.50
H33B	0.4498	0.1373	0.0320	0.146*	0.50
C29B	0.370 (2)	0.2433 (15)	−0.0391 (10)	0.122 (2)	0.50
H29C	0.4776	0.2706	−0.0222	0.146*	0.50
H29D	0.3509	0.3136	−0.0469	0.146*	0.50
C30B	0.317 (3)	0.1273 (16)	−0.1317 (11)	0.122 (2)	0.50
H30C	0.2114	0.1038	−0.1520	0.146*	0.50
H30D	0.3737	0.1487	−0.1790	0.146*	0.50
C31B	0.341 (3)	0.0201 (16)	−0.1169 (10)	0.122 (2)	0.50
H31C	0.2969	−0.0548	−0.1731	0.146*	0.50
H31D	0.4482	0.0400	−0.1091	0.146*	0.50
C32B	0.283 (3)	−0.0146 (17)	−0.0404 (9)	0.122 (2)	0.50
H32C	0.3212	−0.0761	−0.0331	0.146*	0.50
H32D	0.1743	−0.0555	−0.0545	0.146*	0.50
C33B	0.324 (3)	0.0971 (18)	0.0484 (11)	0.122 (2)	0.50
H33C	0.4304	0.1255	0.0705	0.146*	0.50
H33D	0.2681	0.0713	0.0945	0.146*	0.50

### Atomic displacement parameters (Å^2^)

**Table:**

	*U*^11^	*U*^22^	*U*^33^	*U*^12^	*U*^13^	*U*^23^
Sn	0.0761 (2)	0.04922 (19)	0.0596 (2)	0.02397 (15)	0.00460 (15)	0.01359 (14)
O1	0.093 (2)	0.0494 (16)	0.0617 (18)	0.0339 (16)	0.0004 (16)	0.0063 (14)
O2	0.114 (3)	0.063 (2)	0.064 (2)	0.046 (2)	−0.0145 (19)	0.0064 (16)
N1	0.129 (4)	0.063 (3)	0.059 (2)	0.051 (3)	−0.018 (2)	−0.0025 (19)
C1	0.068 (3)	0.049 (2)	0.049 (2)	0.0225 (19)	0.0083 (19)	0.0147 (18)
C2	0.080 (3)	0.048 (2)	0.052 (2)	0.024 (2)	0.006 (2)	0.0105 (18)
C3	0.114 (4)	0.056 (3)	0.064 (3)	0.039 (3)	0.001 (3)	0.013 (2)
C4	0.135 (5)	0.071 (3)	0.080 (4)	0.051 (3)	−0.013 (3)	0.023 (3)
C5	0.140 (5)	0.075 (4)	0.069 (3)	0.045 (4)	−0.025 (3)	0.016 (3)
C6	0.099 (4)	0.059 (3)	0.052 (3)	0.029 (3)	0.001 (2)	0.007 (2)
C7	0.071 (3)	0.050 (2)	0.054 (2)	0.024 (2)	0.008 (2)	0.0098 (19)
C8	0.099 (4)	0.056 (3)	0.056 (3)	0.036 (3)	−0.011 (3)	0.002 (2)
C9	0.133 (6)	0.071 (4)	0.084 (4)	0.024 (4)	0.013 (4)	0.018 (3)
C10	0.142 (6)	0.059 (3)	0.113 (6)	0.010 (4)	−0.012 (5)	0.015 (4)
C11	0.142 (6)	0.068 (4)	0.085 (4)	0.049 (4)	−0.030 (4)	−0.008 (3)
C12	0.099 (4)	0.090 (4)	0.066 (3)	0.052 (4)	−0.012 (3)	−0.002 (3)
C13	0.082 (3)	0.069 (3)	0.072 (3)	0.034 (3)	−0.012 (3)	0.005 (2)
C14	0.140 (7)	0.189 (9)	0.102 (6)	0.098 (7)	0.017 (5)	0.005 (6)
C15	0.088 (4)	0.106 (5)	0.124 (6)	0.018 (4)	0.013 (4)	0.025 (4)
C16	0.079 (3)	0.086 (4)	0.109 (5)	0.033 (3)	0.012 (3)	0.043 (4)
C19	0.084 (2)	0.127 (3)	0.165 (4)	0.040 (2)	0.014 (3)	0.064 (3)
C17A	0.084 (2)	0.127 (3)	0.165 (4)	0.040 (2)	0.014 (3)	0.064 (3)
C18A	0.084 (2)	0.127 (3)	0.165 (4)	0.040 (2)	0.014 (3)	0.064 (3)
C20A	0.084 (2)	0.127 (3)	0.165 (4)	0.040 (2)	0.014 (3)	0.064 (3)
C21A	0.084 (2)	0.127 (3)	0.165 (4)	0.040 (2)	0.014 (3)	0.064 (3)
C17B	0.084 (2)	0.127 (3)	0.165 (4)	0.040 (2)	0.014 (3)	0.064 (3)
C18B	0.084 (2)	0.127 (3)	0.165 (4)	0.040 (2)	0.014 (3)	0.064 (3)
C20B	0.084 (2)	0.127 (3)	0.165 (4)	0.040 (2)	0.014 (3)	0.064 (3)
C21B	0.084 (2)	0.127 (3)	0.165 (4)	0.040 (2)	0.014 (3)	0.064 (3)
C22	0.101 (4)	0.071 (3)	0.077 (3)	0.026 (3)	0.014 (3)	0.025 (3)
C25	0.179 (7)	0.136 (4)	0.112 (3)	0.021 (4)	0.057 (4)	0.050 (3)
C23A	0.179 (7)	0.136 (4)	0.112 (3)	0.021 (4)	0.057 (4)	0.050 (3)
C24A	0.179 (7)	0.136 (4)	0.112 (3)	0.021 (4)	0.057 (4)	0.050 (3)
C26A	0.179 (7)	0.136 (4)	0.112 (3)	0.021 (4)	0.057 (4)	0.050 (3)
C27A	0.179 (7)	0.136 (4)	0.112 (3)	0.021 (4)	0.057 (4)	0.050 (3)
C23B	0.179 (7)	0.136 (4)	0.112 (3)	0.021 (4)	0.057 (4)	0.050 (3)
C24B	0.179 (7)	0.136 (4)	0.112 (3)	0.021 (4)	0.057 (4)	0.050 (3)
C26B	0.179 (7)	0.136 (4)	0.112 (3)	0.021 (4)	0.057 (4)	0.050 (3)
C27B	0.179 (7)	0.136 (4)	0.112 (3)	0.021 (4)	0.057 (4)	0.050 (3)
C28	0.112 (4)	0.056 (3)	0.068 (3)	0.034 (3)	0.019 (3)	0.011 (2)
C29A	0.228 (6)	0.086 (3)	0.072 (3)	0.084 (3)	0.038 (4)	0.029 (2)
C30A	0.228 (6)	0.086 (3)	0.072 (3)	0.084 (3)	0.038 (4)	0.029 (2)
C31A	0.228 (6)	0.086 (3)	0.072 (3)	0.084 (3)	0.038 (4)	0.029 (2)
C32A	0.228 (6)	0.086 (3)	0.072 (3)	0.084 (3)	0.038 (4)	0.029 (2)
C33A	0.228 (6)	0.086 (3)	0.072 (3)	0.084 (3)	0.038 (4)	0.029 (2)
C29B	0.228 (6)	0.086 (3)	0.072 (3)	0.084 (3)	0.038 (4)	0.029 (2)
C30B	0.228 (6)	0.086 (3)	0.072 (3)	0.084 (3)	0.038 (4)	0.029 (2)
C31B	0.228 (6)	0.086 (3)	0.072 (3)	0.084 (3)	0.038 (4)	0.029 (2)
C32B	0.228 (6)	0.086 (3)	0.072 (3)	0.084 (3)	0.038 (4)	0.029 (2)
C33B	0.228 (6)	0.086 (3)	0.072 (3)	0.084 (3)	0.038 (4)	0.029 (2)

### Geometric parameters (Å, °)

**Table:**

Sn—O1	2.073 (3)	C21B—H21C	0.9700
Sn—C16	2.130 (6)	C21B—H21D	0.9700
Sn—C28	2.147 (5)	C22—C27B	1.43 (2)
Sn—C22	2.153 (6)	C22—C23A	1.51 (2)
O1—C7	1.300 (5)	C22—C27A	1.526 (15)
O2—C7	1.228 (5)	C22—C23B	1.527 (17)
N1—C2	1.362 (6)	C22—H22	0.9800
N1—C8	1.425 (6)	C25—C24B	1.464 (15)
N1—H1	0.90 (7)	C25—C24A	1.478 (16)
C1—C6	1.405 (6)	C25—C26B	1.510 (15)
C1—C2	1.417 (6)	C25—C26A	1.519 (16)
C1—C7	1.460 (6)	C25—H25A	0.9700
C2—C3	1.390 (7)	C25—H25B	0.9700
C3—C4	1.360 (8)	C23A—C24A	1.57 (3)
C3—H3	0.9300	C23A—H23A	0.9700
C4—C5	1.375 (8)	C23A—H23B	0.9700
C4—H4	0.9300	C24A—H24A	0.9700
C5—C6	1.356 (8)	C24A—H24B	0.9700
C5—H5	0.9300	C26A—C27A	1.531 (16)
C6—H6	0.9300	C26A—H26A	0.9700
C8—C13	1.366 (8)	C26A—H26B	0.9700
C8—C9	1.376 (9)	C27A—H27A	0.9700
C9—C10	1.364 (9)	C27A—H27B	0.9700
C9—H9	0.9300	C23B—C24B	1.505 (17)
C10—C11	1.362 (11)	C23B—H23C	0.9700
C10—H10	0.9300	C23B—H23D	0.9700
C11—C12	1.346 (10)	C24B—H24C	0.9700
C11—H11	0.9300	C24B—H24D	0.9700
C12—C13	1.417 (7)	C26B—C27B	1.531 (16)
C12—C14	1.499 (11)	C26B—H26C	0.9700
C13—C15	1.485 (9)	C26B—H26D	0.9700
C14—H14A	0.9600	C27B—H27C	0.9700
C14—H14B	0.9600	C27B—H27D	0.9700
C14—H14C	0.9600	C28—C33A	1.465 (15)
C15—H15A	0.9600	C28—C29B	1.498 (14)
C15—H15B	0.9600	C28—C29A	1.539 (14)
C15—H15C	0.9600	C28—C33B	1.576 (14)
C16—C17B	1.461 (16)	C28—H28	0.9800
C16—C21A	1.503 (17)	C29A—C30A	1.500 (15)
C16—C21B	1.527 (17)	C29A—H29A	0.9700
C16—C17A	1.560 (15)	C29A—H29B	0.9700
C16—H16	0.9800	C30A—C31A	1.513 (14)
C19—C20A	1.477 (16)	C30A—H30A	0.9700
C19—C18A	1.483 (15)	C30A—H30B	0.9700
C19—C18B	1.525 (14)	C31A—C32A	1.429 (14)
C19—C20B	1.534 (16)	C31A—H31A	0.9700
C19—H19A	0.9700	C31A—H31B	0.9700
C19—H19B	0.9700	C32A—C33A	1.52 (2)
C17A—C18A	1.557 (18)	C32A—H32A	0.9700
C17A—H17A	0.9700	C32A—H32B	0.9700
C17A—H17B	0.9700	C33A—H33A	0.9700
C18A—H18A	0.9700	C33A—H33B	0.9700
C18A—H18B	0.9700	C29B—C30B	1.546 (15)
C20A—C21A	1.535 (17)	C29B—H29C	0.9700
C20A—H20A	0.9700	C29B—H29D	0.9700
C20A—H20B	0.9700	C30B—C31B	1.474 (12)
C21A—H21A	0.9700	C30B—H30C	0.9700
C21A—H21B	0.9700	C30B—H30D	0.9700
C17B—C18B	1.553 (16)	C31B—C32B	1.442 (15)
C17B—H17C	0.9700	C31B—H31C	0.9700
C17B—H17D	0.9700	C31B—H31D	0.9700
C18B—H18C	0.9700	C32B—C33B	1.488 (16)
C18B—H18D	0.9700	C32B—H32C	0.9700
C20B—C21B	1.540 (17)	C32B—H32D	0.9700
C20B—H20C	0.9700	C33B—H33C	0.9700
C20B—H20D	0.9700	C33B—H33D	0.9700
			
O1—Sn—C16	112.38 (18)	C27A—C22—Sn	117.5 (7)
O1—Sn—C28	94.52 (17)	C23B—C22—Sn	109.6 (9)
C16—Sn—C28	117.4 (3)	C27B—C22—H22	125.4
O1—Sn—C22	105.4 (2)	C23A—C22—H22	97.1
C16—Sn—C22	112.4 (2)	C27A—C22—H22	97.1
C28—Sn—C22	112.7 (2)	C23B—C22—H22	119.7
C7—O1—Sn	111.7 (3)	Sn—C22—H22	97.1
C2—N1—C8	124.6 (4)	C24B—C25—C26B	123.4 (13)
C2—N1—H1	115 (4)	C24A—C25—C26B	117.1 (14)
C8—N1—H1	120 (4)	C24B—C25—C26A	110.5 (14)
C6—C1—C2	117.8 (4)	C24A—C25—C26A	93.7 (15)
C6—C1—C7	120.1 (4)	C24B—C25—H25A	120.1
C2—C1—C7	122.0 (4)	C24A—C25—H25A	113.0
N1—C2—C3	121.2 (4)	C26B—C25—H25A	115.0
N1—C2—C1	120.5 (4)	C26A—C25—H25A	113.0
C3—C2—C1	118.2 (4)	C24B—C25—H25B	87.4
C4—C3—C2	121.3 (5)	C24A—C25—H25B	113.0
C4—C3—H3	119.4	C26B—C25—H25B	85.3
C2—C3—H3	119.4	C26A—C25—H25B	113.0
C3—C4—C5	121.6 (5)	H25A—C25—H25B	110.4
C3—C4—H4	119.2	C22—C23A—C24A	111.2 (19)
C5—C4—H4	119.2	C22—C23A—H23A	109.4
C6—C5—C4	118.3 (5)	C24A—C23A—H23A	109.4
C6—C5—H5	120.8	C22—C23A—H23B	109.4
C4—C5—H5	120.8	C24A—C23A—H23B	109.4
C5—C6—C1	122.7 (5)	H23A—C23A—H23B	108.0
C5—C6—H6	118.6	C25—C24A—C23A	108.5 (17)
C1—C6—H6	118.6	C25—C24A—H24A	110.0
O2—C7—O1	119.8 (4)	C23A—C24A—H24A	110.0
O2—C7—C1	123.5 (4)	C25—C24A—H24B	110.0
O1—C7—C1	116.7 (4)	C23A—C24A—H24B	110.0
C13—C8—C9	121.3 (5)	H24A—C24A—H24B	108.4
C13—C8—N1	119.2 (5)	C25—C26A—C27A	118.1 (15)
C9—C8—N1	119.5 (6)	C25—C26A—H26A	107.8
C10—C9—C8	119.1 (7)	C27A—C26A—H26A	107.8
C10—C9—H9	120.4	C25—C26A—H26B	107.8
C8—C9—H9	120.4	C27A—C26A—H26B	107.8
C11—C10—C9	120.1 (7)	H26A—C26A—H26B	107.1
C11—C10—H10	119.9	C22—C27A—C26A	104.8 (14)
C9—C10—H10	119.9	C22—C27A—H27A	110.8
C12—C11—C10	122.0 (6)	C26A—C27A—H27A	110.8
C12—C11—H11	119.0	C22—C27A—H27B	110.8
C10—C11—H11	119.0	C26A—C27A—H27B	110.8
C11—C12—C13	118.6 (6)	H27A—C27A—H27B	108.9
C11—C12—C14	120.5 (7)	C24B—C23B—C22	115.1 (17)
C13—C12—C14	120.8 (7)	C24B—C23B—H23C	108.5
C8—C13—C12	118.7 (6)	C22—C23B—H23C	108.5
C8—C13—C15	121.2 (5)	C24B—C23B—H23D	108.5
C12—C13—C15	120.1 (6)	C22—C23B—H23D	108.5
C12—C14—H14A	109.5	H23C—C23B—H23D	107.5
C12—C14—H14B	109.5	C25—C24B—C23B	109.0 (17)
H14A—C14—H14B	109.5	C25—C24B—H24C	109.9
C12—C14—H14C	109.5	C23B—C24B—H24C	109.9
H14A—C14—H14C	109.5	C25—C24B—H24D	109.9
H14B—C14—H14C	109.5	C23B—C24B—H24D	109.9
C13—C15—H15A	109.5	H24C—C24B—H24D	108.3
C13—C15—H15B	109.5	C25—C26B—C27B	106.1 (14)
H15A—C15—H15B	109.5	C25—C26B—H26C	110.5
C13—C15—H15C	109.5	C27B—C26B—H26C	110.5
H15A—C15—H15C	109.5	C25—C26B—H26D	110.5
H15B—C15—H15C	109.5	C27B—C26B—H26D	110.5
C17B—C16—C21A	119 (2)	H26C—C26B—H26D	108.7
C17B—C16—C21B	115.7 (19)	C22—C27B—C26B	121.4 (18)
C21A—C16—C17A	110 (2)	C22—C27B—H27C	107.0
C21B—C16—C17A	107 (2)	C26B—C27B—H27C	107.0
C17B—C16—Sn	111.2 (9)	C22—C27B—H27D	107.0
C21A—C16—Sn	114.0 (11)	C26B—C27B—H27D	107.0
C21B—C16—Sn	119.1 (10)	H27C—C27B—H27D	106.7
C17A—C16—Sn	115.2 (8)	C33A—C28—C29B	93.9 (10)
C17B—C16—H16	99.3	C33A—C28—C29A	111.0 (9)
C21A—C16—H16	105.4	C29B—C28—C33B	108.4 (9)
C21B—C16—H16	103.0	C29A—C28—C33B	125.4 (9)
C17A—C16—H16	105.4	C33A—C28—Sn	118.3 (9)
Sn—C16—H16	105.4	C29B—C28—Sn	112.6 (7)
C20A—C19—C18A	110.5 (14)	C29A—C28—Sn	112.6 (7)
C20A—C19—C18B	121.2 (14)	C33B—C28—Sn	109.8 (7)
C18A—C19—C20B	103.6 (14)	C33A—C28—H28	104.4
C18B—C19—C20B	118.9 (13)	C29B—C28—H28	123.6
C20A—C19—H19A	109.5	C29A—C28—H28	104.4
C18A—C19—H19A	109.5	C33B—C28—H28	96.6
C18B—C19—H19A	117.6	Sn—C28—H28	104.4
C20B—C19—H19A	101.4	C30A—C29A—C28	112.1 (12)
C20A—C19—H19B	109.5	C30A—C29A—H29A	109.2
C18A—C19—H19B	109.5	C28—C29A—H29A	109.2
C18B—C19—H19B	87.6	C30A—C29A—H29B	109.2
C20B—C19—H19B	123.9	C28—C29A—H29B	109.2
H19A—C19—H19B	108.1	H29A—C29A—H29B	107.9
C18A—C17A—C16	105.3 (15)	C29A—C30A—C31A	114.3 (13)
C18A—C17A—H17A	110.7	C29A—C30A—H30A	108.7
C16—C17A—H17A	110.7	C31A—C30A—H30A	108.7
C18A—C17A—H17B	110.7	C29A—C30A—H30B	108.7
C16—C17A—H17B	110.7	C31A—C30A—H30B	108.7
H17A—C17A—H17B	108.8	H30A—C30A—H30B	107.6
C19—C18A—C17A	104.7 (15)	C32A—C31A—C30A	108.4 (13)
C19—C18A—H18A	110.8	C32A—C31A—H31A	110.0
C17A—C18A—H18A	110.8	C30A—C31A—H31A	110.0
C19—C18A—H18B	110.8	C32A—C31A—H31B	110.0
C17A—C18A—H18B	110.8	C30A—C31A—H31B	110.0
H18A—C18A—H18B	108.9	H31A—C31A—H31B	108.4
C19—C20A—C21A	119 (2)	C31A—C32A—C33A	111.3 (15)
C19—C20A—H20A	107.5	C31A—C32A—H32A	109.4
C21A—C20A—H20A	107.5	C33A—C32A—H32A	109.4
C19—C20A—H20B	107.5	C31A—C32A—H32B	109.4
C21A—C20A—H20B	107.5	C33A—C32A—H32B	109.4
H20A—C20A—H20B	107.0	H32A—C32A—H32B	108.0
C16—C21A—C20A	112 (2)	C28—C33A—C32A	112.1 (16)
C16—C21A—H21A	109.1	C28—C33A—H33A	109.2
C20A—C21A—H21A	109.1	C32A—C33A—H33A	109.2
C16—C21A—H21B	109.1	C28—C33A—H33B	109.2
C20A—C21A—H21B	109.1	C32A—C33A—H33B	109.2
H21A—C21A—H21B	107.9	H33A—C33A—H33B	107.9
C16—C17B—C18B	119.5 (15)	C28—C29B—C30B	111.6 (13)
C16—C17B—H17C	107.4	C28—C29B—H29C	109.3
C18B—C17B—H17C	107.4	C30B—C29B—H29C	109.3
C16—C17B—H17D	107.4	C28—C29B—H29D	109.3
C18B—C17B—H17D	107.4	C30B—C29B—H29D	109.3
H17C—C17B—H17D	107.0	H29C—C29B—H29D	108.0
C19—C18B—C17B	114.2 (13)	C31B—C30B—C29B	108.2 (12)
C19—C18B—H18C	108.7	C31B—C30B—H30C	110.1
C17B—C18B—H18C	108.7	C29B—C30B—H30C	110.1
C19—C18B—H18D	108.7	C31B—C30B—H30D	110.1
C17B—C18B—H18D	108.7	C29B—C30B—H30D	110.1
H18C—C18B—H18D	107.6	H30C—C30B—H30D	108.4
C19—C20B—C21B	107 (2)	C32B—C31B—C30B	118.5 (14)
C19—C20B—H20C	110.3	C32B—C31B—H31C	107.7
C21B—C20B—H20C	110.3	C30B—C31B—H31C	107.7
C19—C20B—H20D	110.3	C32B—C31B—H31D	107.7
C21B—C20B—H20D	110.3	C30B—C31B—H31D	107.7
H20C—C20B—H20D	108.5	H31C—C31B—H31D	107.1
C16—C21B—C20B	113 (2)	C31B—C32B—C33B	112.9 (13)
C16—C21B—H21C	109.0	C31B—C32B—H32C	109.0
C20B—C21B—H21C	109.0	C33B—C32B—H32C	109.0
C16—C21B—H21D	109.0	C31B—C32B—H32D	109.0
C20B—C21B—H21D	109.0	C33B—C32B—H32D	109.0
H21C—C21B—H21D	107.8	H32C—C32B—H32D	107.8
C27B—C22—C23A	108.1 (14)	C32B—C33B—C28	111.8 (12)
C23A—C22—C27A	121.5 (13)	C32B—C33B—H33C	109.3
C27B—C22—C23B	93.1 (14)	C28—C33B—H33C	109.3
C27A—C22—C23B	114.6 (13)	C32B—C33B—H33D	109.3
C27B—C22—Sn	112.3 (9)	C28—C33B—H33D	109.3
C23A—C22—Sn	116.5 (10)	H33C—C33B—H33D	107.9
			
C16—Sn—O1—C7	−55.7 (4)	C16—Sn—C22—C27B	−84.3 (11)
C28—Sn—O1—C7	−177.9 (3)	C28—Sn—C22—C27B	51.1 (11)
C22—Sn—O1—C7	67.1 (4)	O1—Sn—C22—C23A	−81.6 (13)
C8—N1—C2—C3	4.6 (9)	C16—Sn—C22—C23A	41.1 (13)
C8—N1—C2—C1	−174.8 (5)	C28—Sn—C22—C23A	176.6 (13)
C6—C1—C2—N1	−178.5 (5)	O1—Sn—C22—C27A	121.8 (12)
C7—C1—C2—N1	4.8 (7)	C16—Sn—C22—C27A	−115.5 (12)
C6—C1—C2—C3	2.0 (7)	C28—Sn—C22—C27A	20.0 (13)
C7—C1—C2—C3	−174.6 (5)	O1—Sn—C22—C23B	−105.1 (12)
N1—C2—C3—C4	179.2 (6)	C16—Sn—C22—C23B	17.6 (12)
C1—C2—C3—C4	−1.4 (9)	C28—Sn—C22—C23B	153.1 (12)
C2—C3—C4—C5	−0.2 (11)	C27B—C22—C23A—C24A	−55 (2)
C3—C4—C5—C6	1.0 (11)	C27A—C22—C23A—C24A	−27 (3)
C4—C5—C6—C1	−0.3 (10)	C23B—C22—C23A—C24A	−106 (5)
C2—C1—C6—C5	−1.3 (8)	Sn—C22—C23A—C24A	177.1 (13)
C7—C1—C6—C5	175.5 (6)	C24B—C25—C24A—C23A	56 (3)
Sn—O1—C7—O2	4.3 (6)	C26B—C25—C24A—C23A	−55 (2)
Sn—O1—C7—C1	−173.5 (3)	C26A—C25—C24A—C23A	−75.2 (18)
C6—C1—C7—O2	−175.3 (5)	C22—C23A—C24A—C25	57 (2)
C2—C1—C7—O2	1.3 (7)	C24B—C25—C26A—C27A	59 (2)
C6—C1—C7—O1	2.5 (7)	C24A—C25—C26A—C27A	79 (2)
C2—C1—C7—O1	179.1 (4)	C26B—C25—C26A—C27A	−63 (2)
C2—N1—C8—C13	95.1 (7)	C27B—C22—C27A—C26A	92 (3)
C2—N1—C8—C9	−87.1 (8)	C23A—C22—C27A—C26A	23 (3)
C13—C8—C9—C10	0.9 (10)	C23B—C22—C27A—C26A	47 (2)
N1—C8—C9—C10	−176.7 (6)	Sn—C22—C27A—C26A	178.1 (11)
C8—C9—C10—C11	2.0 (12)	C25—C26A—C27A—C22	−52 (2)
C9—C10—C11—C12	−4.0 (12)	C27B—C22—C23B—C24B	−73 (2)
C10—C11—C12—C13	2.9 (10)	C23A—C22—C23B—C24B	60 (4)
C10—C11—C12—C14	−178.8 (7)	C27A—C22—C23B—C24B	−53 (2)
C9—C8—C13—C12	−2.0 (8)	Sn—C22—C23B—C24B	172.4 (16)
N1—C8—C13—C12	175.7 (5)	C24A—C25—C24B—C23B	−107 (4)
C9—C8—C13—C15	177.5 (6)	C26B—C25—C24B—C23B	−23 (3)
N1—C8—C13—C15	−4.8 (8)	C26A—C25—C24B—C23B	−54 (2)
C11—C12—C13—C8	0.1 (8)	C22—C23B—C24B—C25	54 (3)
C14—C12—C13—C8	−178.2 (6)	C24B—C25—C26B—C27B	18 (3)
C11—C12—C13—C15	−179.4 (6)	C24A—C25—C26B—C27B	48 (2)
C14—C12—C13—C15	2.3 (9)	C26A—C25—C26B—C27B	90 (3)
O1—Sn—C16—C17B	−167.1 (14)	C23A—C22—C27B—C26B	54 (2)
C28—Sn—C16—C17B	−59.1 (15)	C27A—C22—C27B—C26B	−69 (3)
C22—Sn—C16—C17B	74.1 (15)	C23B—C22—C27B—C26B	71 (2)
O1—Sn—C16—C21A	−29 (3)	Sn—C22—C27B—C26B	−176.1 (15)
C28—Sn—C16—C21A	79 (3)	C25—C26B—C27B—C22	−48 (3)
C22—Sn—C16—C21A	−148 (3)	O1—Sn—C28—C33A	167.8 (12)
O1—Sn—C16—C21B	−29 (3)	C16—Sn—C28—C33A	49.7 (12)
C28—Sn—C16—C21B	79 (3)	C22—Sn—C28—C33A	−83.4 (12)
C22—Sn—C16—C21B	−147 (3)	O1—Sn—C28—C29B	59.9 (9)
O1—Sn—C16—C17A	−158.0 (13)	C16—Sn—C28—C29B	−58.2 (9)
C28—Sn—C16—C17A	−49.9 (14)	C22—Sn—C28—C29B	168.7 (9)
C22—Sn—C16—C17A	83.3 (13)	O1—Sn—C28—C29A	36.1 (10)
C17B—C16—C17A—C18A	−98 (12)	C16—Sn—C28—C29A	−82.0 (10)
C21A—C16—C17A—C18A	64 (3)	C22—Sn—C28—C29A	144.9 (10)
C21B—C16—C17A—C18A	60 (3)	O1—Sn—C28—C33B	−179.2 (11)
Sn—C16—C17A—C18A	−164.7 (11)	C16—Sn—C28—C33B	62.7 (11)
C20A—C19—C18A—C17A	63.2 (17)	C22—Sn—C28—C33B	−70.4 (11)
C18B—C19—C18A—C17A	−60 (3)	C33A—C28—C29A—C30A	46 (2)
C20B—C19—C18A—C17A	76.4 (16)	C29B—C28—C29A—C30A	87 (3)
C16—C17A—C18A—C19	−73.9 (19)	C33B—C28—C29A—C30A	43 (2)
C18A—C19—C20A—C21A	−45 (3)	Sn—C28—C29A—C30A	−178.4 (12)
C18B—C19—C20A—C21A	−24 (4)	C28—C29A—C30A—C31A	−48 (2)
C20B—C19—C20A—C21A	−108 (10)	C29A—C30A—C31A—C32A	55 (2)
C17B—C16—C21A—C20A	−40 (5)	C30A—C31A—C32A—C33A	−60 (2)
C17A—C16—C21A—C20A	−43 (5)	C29B—C28—C33A—C32A	−66.4 (18)
Sn—C16—C21A—C20A	−175 (3)	C29A—C28—C33A—C32A	−52 (2)
C19—C20A—C21A—C16	34 (6)	C33B—C28—C33A—C32A	118 (9)
C21A—C16—C17B—C18B	35 (4)	Sn—C28—C33A—C32A	175.2 (11)
C21B—C16—C17B—C18B	31 (4)	C31A—C32A—C33A—C28	62 (2)
C17A—C16—C17B—C18B	54 (10)	C33A—C28—C29B—C30B	57.8 (16)
Sn—C16—C17B—C18B	170.8 (19)	C29A—C28—C29B—C30B	−85 (3)
C18A—C19—C18B—C17B	81 (4)	C33B—C28—C29B—C30B	59.0 (18)
C20B—C19—C18B—C17B	32 (2)	Sn—C28—C29B—C30B	−179.2 (10)
C16—C17B—C18B—C19	−21 (3)	C28—C29B—C30B—C31B	−56 (2)
C20A—C19—C20B—C21B	53 (7)	C29B—C30B—C31B—C32B	51 (3)
C18A—C19—C20B—C21B	−67 (2)	C30B—C31B—C32B—C33B	−49 (3)
C18B—C19—C20B—C21B	−50 (3)	C31B—C32B—C33B—C28	49 (2)
C17B—C16—C21B—C20B	−51 (5)	C33A—C28—C33B—C32B	−50 (7)
C17A—C16—C21B—C20B	−55 (5)	C29B—C28—C33B—C32B	−55 (2)
Sn—C16—C21B—C20B	172 (2)	C29A—C28—C33B—C32B	−39 (3)
C19—C20B—C21B—C16	58 (5)	Sn—C28—C33B—C32B	−178.2 (14)
O1—Sn—C22—C27B	152.9 (11)		

### Hydrogen-bond geometry (Å, °)

**Table:**

*D*—H···*A*	*D*—H	H···*A*	*D*···*A*	*D*—H···*A*
N1—H1···O2	0.90 (7)	1.93 (7)	2.656 (6)	137 (6)
C23A—H23A···O2	0.97	2.45	3.17 (3)	132
